# Epidemiology of nausea and vomiting of pregnancy: prevalence, severity, determinants, and the importance of race/ethnicity

**DOI:** 10.1186/1471-2393-9-26

**Published:** 2009-07-02

**Authors:** Anaïs Lacasse, Evelyne Rey, Ema Ferreira, Caroline Morin, Anick Bérard

**Affiliations:** 1 Faculty of Pharmacy, University of Montreal, Montreal, Quebec, Canada; 2 Research Center, CHU Sainte-Justine, Montreal, Quebec, Canada; 3 Department of Obstetrics & Gynecology, CHU Sainte-Justine, Montreal, Quebec, Canada; 4 Faculty of Medicine, University of Montreal, Montreal, Quebec, Canada; 5 Department of Pharmacy, CHU Sainte-Justine, Montreal, Quebec, Canada

## Abstract

**Background:**

Studies that contributed to the epidemiology of nausea and vomiting of pregnancy have reported conflicting findings, and often failed to account for all possible co-variables necessary to evaluate the multidimensional associations. The objectives of this study were to: 1) Estimate the prevalence and the severity of nausea and vomiting of pregnancy during the 1^st ^and the 2^nd ^trimester of pregnancy, and 2) Identify determinants of presence and severity of nausea and vomiting of pregnancy during the 1^st ^and 2^nd ^trimesters separately, with a special emphasis on the impact of race/ethnicity.

**Methods:**

A prospective study including pregnant women attending the Centre Hospitalier Universitaire (CHU) Sainte-Justine or René-Laennec clinics for their prenatal care was conducted from 2004 to 2006. Women were eligible if they were ≥ 18 years of age, and ≤ 16 weeks of gestation. Women were asked to fill out a 1^st ^trimester self-administered questionnaire and were interviewed over the telephone during their 2^nd ^trimester of pregnancy. Presence of nausea and vomiting of pregnancy was based on the reporting of pregnant women (yes/no); severity of symptoms was measured by the validated modified-PUQE index.

**Results:**

Of the 367 women included in the study, 81.2% were Caucasians, 10.1% Blacks, 4.6% Hispanics, and 4.1% Asians. Multivariate analyses showed that race/ethnicity was significantly associated with a decreased likelihood of reporting nausea and vomiting of pregnancy (Asians vs. Caucasians OR: 0.13; 95%CI 0.02–0.73; and Blacks vs. Caucasians OR: 0.29; 95%CI 0.09–0.99).

**Conclusion:**

Our study showed that race/ethnicity was associated with the reporting of nausea and vomiting of pregnancy in the 1^st ^trimester of pregnancy.

## Background

Fifty to ninety percent of pregnant women experience nausea and vomiting during their first trimester [1]. In general nausea and vomiting of pregnancy (NVP) appear between the 4^th ^and 6^th ^week of gestational age, with a peak observed between week 8 and 12 [2,3]. A more severe form of NVP, known as *hyperemesis gravidarum *(HG), will also occur in 0.5 to 3 percent of pregnancies and may lead to hospitalisation [4,5]. The aetiology of NVP is poorly understood but NVP could be considered as a multifactorial problem. Proposed theories involve hormonal, vestibular system, gastrointestinal, psychological, hyperolfaction, genetic, and evolutionary factors as possible causes [6,7].

Studies that contributed to the epidemiology of NVP have reported conflicting findings, and often failed to account for all possible co-variables necessary to evaluate the multidimensional associations [8-12]. For example, whether race/ethnicity influence NVP is not well established. While some studies indicate that incidence of NVP differs according to women's race/ethnicity [13-18], others show no association [9,19]. The fact that none of these studies accounted for potential confounders could partly explain this variability. Moreover, studies did not always have an explicit and valid classification of race/ethnicity, and nearly all of them were done on pregnant women with HG. The reason for the possible association between race/ethnicity and NVP is unknown but it has been proposed that differences between racial/ethnic groups could be accounted by important socioeconomic variables [12]. Only three studies used multivariate analyses to evaluate the association between race/ethnicity and NVP and they show contradictory results. The first one failed to demonstrate a significant relationship between race/ethnicity and vomiting [12], the second showed that White women had less NVP than Hispanics and Blacks [11], and the third one showed that Black women had less NVP than whites [20]. The racial/ethnic differences in the reporting of health problems is pivotal in order to find out the reason why patients experience diseases and treatment differently, and to expand approaches for the improvement of public health [21-23]. Therefore, a study looking at the association between race/ethnicity and NVP, accounting for possible confounders is warranted.

The first objective of this study was to estimate the prevalence and the severity of NVP during the 1^st ^and the 2^nd ^trimester of pregnancy according to race/ethnicity. Furthermore, we sought to identify the determinants of NVP during the 1^st ^and the 2^nd ^trimester of pregnancy separately, with a special emphasis on the impact of race/ethnicity.

## Methods

### Study design and study population

We carried out a prospective study of pregnant women receiving prenatal care at the obstetrics and gynaecology outpatient clinic of the *Centre Hospitalier Universitaire Sainte-Justine *(CHU Sainte-Justine) or the *René-Laennec *outpatient clinic, both affiliated with the University of Montreal, Quebec, Canada, from October 2004 to March 2006. Women were eligible if they: 1) were at least 18 years of age; 2) were at their first prenatal visit; 3) were pregnant within 16 weeks of the first day of their last menses; 4) were able to read and understand French or English; and 5) had given their written informed consent. Ethics approval was obtained from the CHU Sainte-Justine's ethics committee. The present study design and data collection were partly described elsewhere [24-27].

### Data collection

At the end of their first prenatal visit, eligible women who accepted to participate were asked to fill out a self-administered questionnaire (1^st ^trimester questionnaire) at home, and return it to the coordinating center at CHU Ste-Justine within the next seven days. Reminder calls were made if self-administered questionnaires were not returned in time. Between weeks 20 and 26 of gestational age, women were called at home (2^nd ^trimester interview) in order to collect additional information. The end of the follow-up was defined as the delivery, where data on pregnancy outcomes were available from CHU Sainte-Justine's medical charts.

Presence and severity of NVP were evaluated in two distinct gestational periods: The 1^st ^trimester questionnaire covered the 1^st ^trimester of pregnancy, and the 2^nd ^trimester interview covered the period between the beginning of the 2^nd ^trimester and the second interview. Presence of NVP was self-reported by women as a dichotomous variable (yes/no). The severity of NVP was measured by the modified-PUQE which has been validated during the whole 1^st ^trimester of pregnancy [24]. This index is based on three physical symptoms: the duration of nausea in hours, and the number of retching and vomiting episodes on an average day since the beginning of pregnancy. The total score can range between 3 and 15, with 3 to 6 representing mild symptoms, 7 to 12 moderate symptoms, and 13 to 15 severe symptoms. The validation study of the modified-PUQE did not support the presence of recall bias in the evaluation of the severity of NVP during the first trimester of pregnancy[24] Furthermore, NVP severity in the 2^nd ^trimester of pregnancy was measured by the modified-PUQE by changing the introduction sentence in order to cover NVP symptoms during this trimester. Intensity of nausea was estimated using a 1 to 10 visual analog scale ("no nausea at all" to "unbearable nausea") in the 1^st ^trimester questionnaire and was verbally self-reported by women on the same scale in the 2^nd ^trimester telephone interview. Women were also asked about excessive salivation experience (spitting). The distress caused by excessive salivation was measured by a 5-point Likert scale.

Demographic and socio-economic data collected in the 1^st ^trimester questionnaire included maternal and gestational age, medication (Rx) insurance plan, work status, living arrangement, education level, household income, and country of birth. Racial/ethnic groups were defined based on self-perceived membership in racial/ethnic groups (White, Chinese, South Asian, Black, Arab/West Asian, Filipino, South East Asian, Latin American, Japanese, Korean, and Other). Because of small frequencies, Chinese, South Asian, South East Asian, and Korean women were regrouped in a broader category. Moreover, as they are often classified as Caucasians [28], Arab women (n = 28) were regrouped with white women (n = 270). We are confident that this was the appropriate decision since prevalence of NVP was similar among women that were clustered in broader categories. Information on lifestyle, co-morbidities, pregnancy history, maternal height, and weight was collected in the 1^st ^trimester questionnaire. Regarding medications used, the 1^st ^trimester questionnaire included vitamins and oral contraceptives (OC) use, and medications use for NVP in the 1^st ^trimester of pregnancy, including non pharmacological methods. In addition to NVP status in the 2^nd ^trimester of pregnancy, data on gestational age, maternal weight gain, and comorbidities during pregnancy were collected.

### Statistical analysis

Descriptive statistics were used to estimate the prevalence and the severity of NVP during the 1^st ^and the 2^nd ^trimester of pregnancy. NVP status in the 1^st ^trimester of pregnancy among racial/ethnic groups were compared using Fisher's exact tests, and ANOVA when appropriate; if significant, additional analyses for specific pairwise comparisons were used. Furthermore, univariate and multivariate logistic regression models were used to identify determinants of NVP in the 1^st ^and the 2^nd ^trimester of pregnancy separately. All socio-demographic variables were included in the multivariate models. Co-variables were selected on the basis of the published literature and their association with the outcome (NVP) in the univariate regression models (p ≤ 0.15 included). In order to evaluate the impact of the presence of NVP in previous pregnancies on NVP in the 1^st ^trimester of subsequent pregnancies, the same multivariate logistic regression model was used in the subgroup of multigravida women. Since foetal sexual differentiation occurs between the 7^th ^and 9^th ^week of gestational age [29], sex of the newborn were included when identifying determinants of NVP in the 2^nd ^trimester of pregnancy. In cohort studies, when the outcome of interest is common, the adjusted odds ratio from the logistic regression may exaggerate a risk association[30] However, correction methods to address this issue can be complex or become inefficient when several variables are included in the model[30] Because identification of predictors is more important than quantification in the case of our study, we did not achieve a correction of odds ratios. Subsequently, univariate and multivariate ordinal regression models were used to identify determinants of NVP severity in the 1^st ^trimester of pregnancy. Medications and non-pharmacological methods used to ease NVP were added in the final model. Significance was computed at p < 0.05. All statistical analyses were performed using SAS Version 8.02 (SAS Institute, NC, USA).

## Results

**Figure 1:**
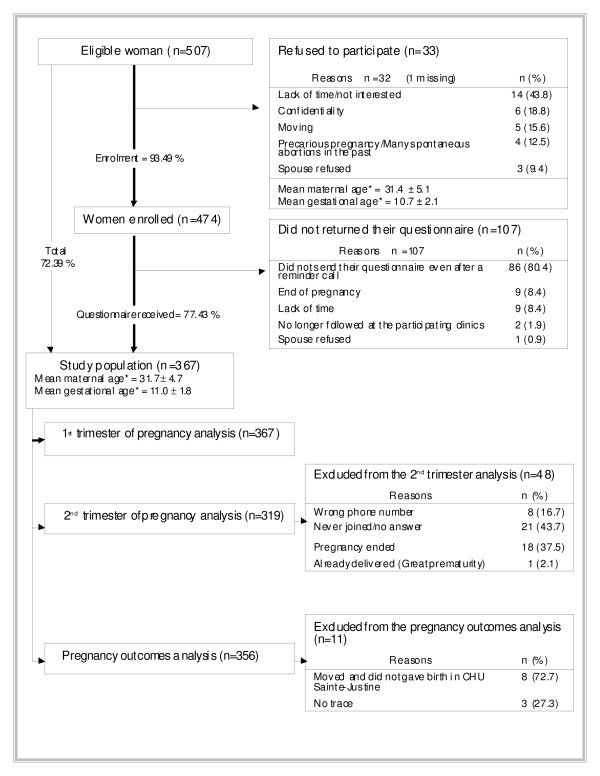
**Study population recruitment and follow-up**. *Maternal and gestational age at inclusion.

A detailed flow-chart of the recruitment, the refusals, and the follow-up is presented in Figure 1. Our study population consisted of 367 pregnant women recruited between 2004 and 2006. Maternal characteristics including race/ethnicity and NVP status of the study population are presented in Table 1. In the 1^st ^trimester of pregnancy, 78.5% of women reported NVP. In this group, 52.2% experienced mild NVP, 45.3% moderate NVP, and 2.5% severe NVP. In addition, 26% of pregnant women reporting NVP also reported excessive salivation during the 1^st ^trimester of pregnancy, and the majority of them were distressed by this situation (Table 1). When asking what women had used in their first trimester to ease nausea and vomiting, 20.4% of them reported having used medications, and 17.9% non-pharmacological methods. As for the 2^nd ^trimester of pregnancy, 40.1% of women reported NVP (data not showed). Among them, 63.3% experienced mild NVP, 35.9% moderate NVP, and 0.8% severe NVP. Intensity of nausea and excessive salivation experience was similar to what had been reported in the 1^st ^trimester of pregnancy. Globally, in our study population, 41.1% of women reported NVP in the 1^st ^trimester of pregnancy only, 1.3% in the 2^nd ^trimester of pregnancy only, and 38.9% in both of these two gestational periods.

**Table 1:** Maternal characteristics and NVP status during pregnancy.

**NVP status n = 367**	**1^st ^trimester of pregnancy **^a^	
**Maternal age-yr **(mean ± SD)	31.74	± 4.70

**Gestational Age -wk **(mean ± SD)	11.03	± 1.84

**Race/ethnicity**- n (%)		
Caucasian	298	(81.20)
Asian	15	(4.09)
Black	37	(10.08)
Hispanic	17	(4.63)

**NVP **- n (%)		
Yes	288	(78.47)
No	79	(21.53)

**Severity of NVP **– n (%)		
Mild	145	(52.16)
Moderate	126	(45.32)
Severe	7	(2.52)

**Intensity of nausea (range 0–10^b^) **– (mean ± SD)	4.69	± 2.48

**Excessive salivation**- n (%)	73	(25.98)

**Distress caused by excessive salivation**- n (%)		
Not at all	7	(9.72)
Slightly	24	(33.33)
Moderatly	17	(23.61)
*Greatly*	7	(9.72)
*Unbearable*	17	(23.61)

**Medications use to treat NVP in 1^st ^trimester**^c ^– n (%)	57	(20.36)

**Non-pharmacological methods use for NVP in 1^st ^trimester**^d^–n (%)	50	(17.86)

NVP = Nausea and vomiting of pregnancy

^a ^Missing values in the 1^st ^trimester questionnaire: Severity of NVP (n = 10); Excessive salivation (n = 7); Distress caused by excessive salivation (n = 1); medications and non-pharmacological methods use for NVP (n = 8).

^b ^0 represented "no nausea at all"and 10 represented "unbearable nausea".

^c ^Medications reported to be used in the 1^st ^trimester of pregnancy to treat NVP were mostly (86%) the combination doxylamine/pyridoxine. Women also reported using metoclopramide, dimenhydrinate, hydroxyzine, meclizine, acetaminophen or acid reflux therapies.

^d ^Non-pharmacological methods used in the 1^st ^trimester of pregnancy included lifestyle and dietary changes, and the use of ginger, acupressure, homeopathic products, herbal teas, mint or lemon.

As showed in Table 2, the reporting of NVP symptoms was not significantly different between racial/ethnic groups (p = 0.06). However, pairwise comparisons showed that NVP was significantly more prevalent in Hispanic (94.1%) women compared to Asians (60%; p < 0.05). Proportion of women reporting excessive salivation in the 1^st ^trimester of pregnancy was higher in Blacks (79.2%) than in Caucasians or Hispanics (Caucasians 19.6%, Hispanics 35.7%; p < 0.05).

**Table 2:** Prevalence and severity of NVP in the 1^st ^trimester of pregnancy according to race/ethnicity.

**Race/ethnicity **n (%)	**Prevalence^a ^n = 288**	**Severity**^b^			**Intensity of nausea**^c^ (mean ± SD)	**Excessive salivation experience**^d^
				
		**Mild n = 145**	**Moderate n = 126**	**Severe n = 7**		
**Caucasian n = 298**	237(79.53)	123 (52.79)	105 (45.06)	5 (2.15)	4.55 ± 2.41	46 (19.57)

**Asian n = 15**	9 (60.00)	4 (50.00)	4 (50.00)	0	5.17 ± 2.79	3 (37.50)

**Black n = 37**	26 (70.27)	11 (45.83)	11 (45.83)	2 (8.33)	5.40 ± 2.88	19 (79.17)

**Hispanic n = 17**	16 (94.12)	7 (53.85)	6 (46.15)	0	5.13 ± 2.68	5 (35.71)

^a ^Fisher's exact test: p = 0.06 (Pairwise comparisons p < 0.05: Asian vs Hispanic)

^b ^Fisher's exact test: p = 0.66

^c ^ANOVA: p = 0.34

^d ^Fisher's exact test: p < 0.0001 (Pairwise comparisons p < 0.05: Black vs Caucasian and Black vs Hispanic)

Multivariate analyses [see Additional file 1] showed that race/ethnicity (Asians vs. Caucasians, and Blacks vs. Caucasians), household income (40000–79999 vs. < 40000 cdn$/yr), and OC use in the six months before pregnancy were significantly associated (p < 0.05) with a decreased likelihood of reporting NVP. In the 303 multigravida women, nausea in previous pregnancies was significantly associated with an increased likelihood to report NVP in the 1^st ^trimester of pregnancy (OR: 3.17; 95%CI 1.25–8.03).

The 2^nd ^trimester interview data were available for 319 of the 367 (87%) women of the study population. Reasons for which women were excluded from the 2^nd ^trimester analysis are presented in Figure 1. Multivariate models (not showed in table form) showed that greater gestational age at the 2^nd ^trimester interview (OR: 0.89; 95%CI 0.79–1.00), exercise during the 1^st ^trimester of pregnancy (OR: 0.49; 95%CI 0.24–0.98), coffee drinking before pregnancy (OR: 0.32; 95%CI 0.12–0.89), and weight gain during the 1^st ^trimester (OR: 0.73; 95%CI 0.61–0.88) were significantly associated with a decreased likelihood of reporting NVP in the 2^nd ^trimester of pregnancy. Presence of NVP during the 1^st ^trimester of pregnancy (OR: 14.80; 95%CI 4.48–48.95), and female foetus gender (OR: 2.08; 95%CI 1.11–3.90) were significantly associated with an increased likelihood of reporting NVP in the 2^nd ^trimester of pregnancy. Race/ethnicity was not a determinant of NVP in the 2^nd ^trimester of pregnancy.

Multivariate analyses [see Additional file 2] showed that being born outside Canada, using medications to ease NVP, using non-pharmacological methods to ease NVP, and parity (2 or more children vs. 0), were significantly associated (p < 0.05) with more severe NVP symptoms during the 1^st ^trimester of pregnancy. Race/ethnicity was not found to be associated with NVP severity in the 1^st ^trimester of pregnancy.

## Discussion

In the 1^st ^trimester of pregnancy, 78.5% of women reported NVP; this prevalence decreased to 40.1% at the beginning of the 2^nd ^trimester of pregnancy. Our study showed that when potential confounders are taken into account, race/ethnicity is associated with the reporting of NVP in the 1^st ^trimester of pregnancy (Black and Asian women being less likely to report NVP than Caucasian women).

### Importance of race/ethnicity

In our study population, prevalence of NVP in the 1^st ^trimester of pregnancy was comparable to what has been reported in the general population (50–90%) [1]. Our data showed that Black and Asian women are less likely to have 1^st ^trimester NVP. Only three previous studies used multivariate analysis when evaluating the association between race/ethnicity and NVP. The first one, conducted in 1985, focused on vomiting symptoms without accounting for nausea, and failed to demonstrate a significant association [12]. The second one (1988), showed that White women had less NVP than non-Whites [11]. Only the third one is not in opposition with our findings, showing that Black women are less likely to have NVP than Whites [20]. Different study populations, study designs, evaluations of NVP symptoms, and confounders considered could partly explain disparity between our results and previous researches. As stated earlier, the etiologic explanation for the racial/ethnic variability in NVP prevalence is unknown. Nevertheless, we are confident that the association between race/ethnicity and 1^st ^trimester NVP found in this study was not due to sociodemographic differences between the groups since potential confounding factors were considered and adjusted for. However, since a relationship between dietary composition and NVP has previously been reported [31], we cannot exclude the possibility of such racial/ethnic differences in our study. Moreover, it is possible that race/ethnicity affects the reporting (ex: different cultural acceptability) of NVP. Globally, we have to point out that our study cannot be definitive in this area because of small frequencies in some of the racial/ethnic groups, and considering the borderline odds ratios for Black women. Although further studies are needed to confirm this result, our findings have some clinical implications regarding specific groups of patients where management of NVP should be optimised.

Excessive salivation was found to be distressful to a majority of pregnant women suffering from NVP. Clinical observations generated the hypothesis that Black women suffering from HG often experience this symptom. As expected, the current study showed that Black women having NVP are more likely to report excessive salivation and spitting in the 1^st ^trimester of pregnancy as compared to Asian, Hispanic, and Caucasian women. We still do not know whether race/ethnicity affects only the reporting of excessive salivation (ex: different cultural acceptability), or if there is a true physiological difference between racial/ethnic groups.

### Other determinants of NVP

It has been previously reported that low socioeconomic status was associated with NVP [6]. Our results suggest that women having a family income ranging from 40000 to 79999 cdn$/y are less likely to have NVP in the 1^st ^trimester of pregnancy than women with lower household income (<40000 cdn$/y). In contrast, a study conducted in 2000 in Quebec, Canada, found that household income ranging from 30000 to 40000 cdn$/y was a determinant of NVP in the 1^st ^trimester of pregnancy (vs. <30 000 or >40 000 cdn$/y) [32]. The different definitions of household income categories make it difficult to do a comparative interpretation of our results, and to establish the generalizability of the association between household income and NVP. Our study also showed that OC use in the six months prior to pregnancy decreased the likelihood of reporting NVP in the 1^st ^trimester. On the other side, an earlier study reported that a longer OC use prior to pregnancy increased NVP severity [31]. However, since our data did not provide a measure of the duration of OC use, a comparison is not possible. Finally, our data are consistent with published findings regarding nausea in previous pregnancies as a predictor of NVP in subsequent pregnancies. [11,32] Few studies have looked at reporting of NVP in the 2^nd ^trimester of pregnancy specifically [33]. In our study, longer gestational age at the 2^nd ^trimester interview was associated with a decreased likelihood of reporting NVP in the 2^nd ^trimester of pregnancy (median gestational age at interview = 21 weeks of pregnancy). Since most of the NVP symptoms disappear by the 20^th ^week of gestation [2], it is normal to see that the more a woman progresses in her pregnancy, the less NVP she will experience. In addition, our results support the reported associations between NVP and smaller weight gain in pregnancy [8] or female fetal gender [10,17]. In contrast with studies in other populations [31,34], we found that coffee drinking before pregnancy and exercise during pregnancy decreased the likelihood of reporting NVP in the 2^nd ^trimester of pregnancy.

As for the severity of NVP, our study suggests that severe NVP symptoms are more common among women who were born outside Canada. This finding is the generalisation of an earlier study who found that women who were born outside Quebec are more likely to have HG, a severe form of NVP [16]. In our study, multiparity was a determinant of more severe NVP symptoms during the 1^st ^trimester of pregnancy, which is consistent with the existing literature [8,35,36]. Medications or non-pharmacological methods use to ease NVP symptoms were also found to be associated with more severe NVP in the 1^st ^trimester of pregnancy. As we can expect, the decision to use medications or non-pharmacological methods to alleviate NVP can be associated with more severe NVP. However, since medications or non-pharmacological methods use can influence or be the consequence of NVP severity, we cannot exclude the possibility of a protopathic bias here.

Globally, discrepancies between our results and what is found in the literature might be partly explained by study population differences, different definitions of covariates and NVP measures, methodological designs, possibility of type I and type II errors, and confounding factors considered in the statistical analysis.

### Strengths and limitations

Because patients experiencing NVP could be more likely to enrol in studies like ours, the study was presented to women as a general evaluation of quality of life during pregnancy without focusing specifically on NVP, thus reducing selection bias. Furthermore, women who refused to participate were comparable to women enrolled in the study regarding maternal and gestational age. Recall bias should be minimised because of the use of a validated NVP questionnaire and because women were asked about recent life-style and health information. However, like in other surveys, there may be under-reporting of smoking and alcohol use during pregnancy in our study since these behaviours are known to affect pregnancy outcome and are considered socially undesirable [37]. Regarding racial/ethnic categorisation, we cannot exclude the possibility of non differential misclassification. Moreover, given that at the time of analyses, clustering of some racial/ethnic groups in broader categories was made, the applicability of our results to underrepresented racial/ethnic groups could be limited. It remains however that race/ethnicity is essential in epidemiologic studies in order to make and explore hypotheses on risk factors and to expand approaches for the improvement of public health [21]. One limitation of our study is that we did not achieve a global 2^nd ^trimester of pregnancy measure of the prevalence and severity of NVP. Because 2^nd ^trimester interview covered the period between the 1^st ^trimester questionnaire and the 2^nd ^trimester interview, we obtained a measure for the period corresponding to the beginning of the 2^nd ^trimester of pregnancy rather than for the entire 2^nd ^trimester. Since NVP symptoms normally disappear by the 20^th ^week of gestation [2], we probably obtained a overestimation of the prevalence and the severity of NVP in the 2^nd ^trimester of pregnancy. Finally, missing values are not a major concern in our study because less than 3% of different maternal characteristic variables were missing.

As for external validity, we feel confident that our study population is representative of the Montreal population. The high degree of racial/ethnic diversity of our region provides a greater generalizability of our results. Moreover, the majority of women in our study population were Caucasians. Consequently, it improves the external validity of our results to the Canadian population. In fact, in 2001 less than 15% of the Canadian population were from a visible minority group [38]. The high education level and the high proportion of women having a household income greater than $80,000/year, which can be explained by the geographic location of the René-Laennec clinic (provided 63% of our study cohort), could however limit the external validity of our study. Finally, because we achieved more than one recruitment site, we are confident that our study cohort is representative of the population of pregnant women receiving prenatal care without an overrepresentation of high-risk pregnancies.

## Conclusion

NVP are a significant health problem in pregnancy. Our study suggests that Black and Asian women have less NVP in the 1^st ^trimester of pregnancy than Caucasians. We also showed that race/ethnicity affects reporting of other conditions associated with NVP like excessive salivation. Our results can contribute to existing literature regarding disease aetiology and are relevant for targeting specific groups of patients such as Caucasian or multiparous where management of NVP should be optimised. In fact, the subject of NVP should be thoroughly investigated in these groups as for the severity of symptoms, the extent to which this condition disturbed everyday tasks, or the need for treatment. Since the association between race/ethnicity and NVP has not been extensively studied, further studies are needed to validate our findings.

## Competing interests

The authors declare that they have no competing interests.

## Authors' contributions

Each author has participated actively and sufficiently in this study, and fulfils all authorship criteria of the International Committee of Medical Journal Editors. AL made substantial contribution to acquisition of data, analysis and interpretation of data, and drafting of the article. ER, EF, and CM made substantial contribution to conception and design of the study and interpretation of data. AB made substantial contribution to conception and design of the study, analysis and interpretation of data, and drafting of the article. Each author revised critically the manuscript and provided final approval of the version to be published.

## Pre-publication history

The pre-publication history for this paper can be accessed here:



## Supplementary Material

Additional file 1 **Table of the determinants of NVP in the 1^st ^trimester of pregnancy**. Multivariate analyses showed that race/ethnicity, household income, and OC use in the six months before pregnancy were significantly associated with a decreased likelihood of reporting NVP. Click here for file

Additional file 2 **Table of the determinants of NVP severity in the 1^st ^trimester of pregnancy**. Multivariate analyses showed that being born outside Canada, using medications to ease NVP, using non-pharmacological methods to ease NVP, and parity, were significantly associated with more severe NVP symptoms during the 1^st ^trimester of pregnancy. Race/ethnicity was not found to be associated with NVP severity in the 1^st ^trimester of pregnancy. Click here for file
