# Supplementary material for: Epidemiology of nausea and vomiting of pregnancy: prevalence, severity, determinants, and the importance of race/ethnicity
Source: BMC Pregnancy Childbirth. 2009 Jul 2;9:26. doi: 10.1186/1471-2393-9-26 (PMC2713199; doi:10.1186/1471-2393-9-26)
Supplement: Additional file 1 — Table of the determinants of NVP in the 1st trimester of pregnancy. Multivariate analyses showed that race/ethnicity, household income, and OC use in the six months before pregnancy were significantly associated with a decreased likelihood of reporting NVP. [file 1471-2393-9-26-S1.doc]

**Additional file 1 - Determinants of NVP in the 1^st^** trimester of pregnancy.

| **Characteristics** | **No NVP**  **n = 79** | | **NVP**  **n = 288** | | **Crude OR**  **(95% CI)** | **Adjusted OR ^a^**  **(95% CI)** |
| --- | --- | --- | --- | --- | --- | --- |
| **Socio-demographics characteristics at 1^st^ trimester questionnaire (**median 11 weeks of pregnancy) | | | | | | |
| **Maternal age– yr** (mean ± SD) | 32.08 | ± 4.99 | 31.65 | ± 4.62 | 0.98 (0.93-1.03) | 0.95 (0.88-1.02) |
| **Gestational age – wk** (mean ± SD) | 11.18 | ± 2.16 | 11.00 | ± 1.75 | 0.95 (0.83-1.08) | 0.91 (0.78-1.07) |
| **Country of birth –** n (%) |  |  |  |  |  |  |
| Canada | 53 | (67.09) | 189 | (65.63) | 1 | 1 |
| Other | 26 | (32.91) | 99 | (34.38) | 1.07 (0.63-1.81) | 1.49 (0.57-3.90) |
| **Race/ethnicity –** n (%) |  |  |  |  |  |  |
| Caucasian | 61 | (77.22) | 237 | (82.29) | 1 | 1 |
| Asian | 6 | (7.59) | 9 | (3.13) | 0.39 (0.13-1.13) | 0.13 (0.02-0.73) |
| Black | 11 | (13.92) | 26 | (9.03) | 0.61 (0.29-1.30) | 0.29 (0.09-0.99) |
| Hispanic | 1 | (1.27) | 16 | (5.56) | 4.12 (0.54-31.66) | 2.36 (0.26-21.61) |
| **Rx insurance plan –** n (%) |  |  |  |  |  |  |
| Provincial plan (RAMQ) only | 18 | (23.38) | 81 | (28.32) | 1 | 1 |
| Other insurance | 59 | (76.62) | 205 | (71.68) | 0.77 (0.43-1.39) | 1.32 (0.55-3.17) |
| **Work status –** n (%) |  |  |  |  |  |  |
| Student or not working | 13 | (16.88) | 72 | (25.09) | 1 | 1 |
| Working | 64 | (83.12) | 215 | (74.91) | 0.61 (0.32-1.17) | 0.56 (0.22-1.42) |
| **Living arrangement –** n (%) |  |  |  |  |  |  |
| With spouse or with someone (family or cotenant) | 76 | (97.44) | 281 | (97.91) | 1 | 1 |
| Living alone | 2 | (2.56) | 6 | (2.09) | 0.81 (0.16-4.10) | 1.63 (0.15-18.31) |
| **Education level–** n (%) |  |  |  |  |  |  |
| University completed | 41 | (53.25) | 185 | (64.46) | 1 | 1 |
| University not completed | 36 | (46.75) | 102 | (35.54) | 0.63 (0.38-1.04) | 0.61 (0.31-1.21) |
| **Household income – cdn$/yr** n (%) |  |  |  |  |  |  |
| Less than 40 000$ | 16 | (21.05) | 88 | (31.43) | 1 | 1 |
| Between 40 000 and 79 999$ | 30 | (39.47) | 70 | (25.00) | 0.42 (0.21-0.84) | 0.35 (0.13-0.93) |
| 80 000 and over $ | 30 | (39.47) | 122 | (43.57) | 0.74 (0.38-1.44) | 0.79 (0.25-2.52) |
| **Lifestyle habits** |  |  |  |  |  |  |
| **Exercise during 1^st^ trimester –** n (%) | 31 | (39.24) | 109 | (38.25) | 0.96 (0.58-1.60) | 0.60 (0.31-1.15) |
| **Smoking before pregnancy–** n (%) | 13 | (16.67) | 37 | (12.85) | 0.74 (0.37-1.47) | 1.07 (0.37-3.05) |
| **Smoking during 1^st^ trimester –** n (%) | 6 | (7.69) | 9 | (3.13) | 0.39 (0.13-1.12 | 0.28 (0.05-1.50) |
| **Caffeine drinking before pregnancy–** n (%) | 66 | (83.54) | 234 | (81.25) | 0.85 (0.44-1.66) | 0.95 (0.38-2.39) |
| **Caffeine drinking during 1^st^ trimester–** n (%) | 46 | (58.23) | 165 | (57.49) | 0.97 (0.59-1.61) | 1.25 (0.61-2.58) |
| **Use of alcohol before pregnancy–** n (%) | 53 | (67.09) | 190 | (65.97) | 0.95 (0.56-1.61) | 1.13 (0.50-2.54) |
| **Use of alcohol during 1^st^ trimester–** n (%) | 12 | (15.19) | 21 | (7.32) | 0.44 (0.21-0.94) | 0.80 (0.28-2.26) |
| **Health status and medications** |  |  |  |  |  |  |
| **Infections or another situation causing nausea and/or vomiting during 1^st^ trimester ^b^–** n (%) | 10 | (12.66) | 46 | (16.14) | 1.33 (0.64-2.77) | 2.03 (0.81-5.13) |
| **Comorbidities before pregnancy ^c^ –** n (%) |  |  |  |  |  |  |
| 0 | 59 | (74.68) | 207 | (71.88) | 1 |  |
| 1 | 18 | (22.78) | 70 | (24.31) | 1.11 (0.61-2.01) |  |
| 2 or 3 | 2 | (2.53) | 11 | (3.82) | 1.57 (0.34-7.26) |  |
| **Vitamin use before pregnancy ^d^–** n (%) | 34 | (43.04) | 111 | (38.54) | 0.83 (0.50-1.38) | 0.78 (0.40-1.52) |
| **Vitamin use during 1^st^ trimester ^d^–** n (%) | 64 | (81.01) | 238 | (83.22) | 1.16 (0.61-2.21) | 1.20 (0.52-2.77) |
| **Oral contraceptives use in the last 6 months before pregnancy –** n (%) |  |  |  |  |  |  |
| No | 60 | (75.95) | 241 | (84.56) | 1 | 1 |
| Yes | 19 | (24.05) | 44 | (15.44) | 0.58 (0.31-1.06) | 0.42 (0.20-0.89) |
| **Pregnancy history** |  |  |  |  |  |  |
| **Gravidity –** n (%) |  |  |  |  |  |  |
| Multigravida | 63 | (79.75) | 240 | (83.33) | 1 |  |
| Primigravida | 16 | (20.25) | 48 | (16.67) | 0.79 (0.42-1.48) |  |
| **Parity –** n (%) |  |  |  |  |  |  |
| 0 | 19 | (24.36) | 70 | (24.48) | 1 | 1 |
| 1 | 41 | (52.56) | 152 | (53.15) | 1.01 (0.55-1.86) | 0.80 (0.35-1.83) |
| 2 or more (max: 4) | 18 | (23.08) | 64 | (22.38) | 0.97 (0.47-2.00) | 0.87(0.33-2.32) |
| **Infertility experience –** n (%) | 6 | (7.79) | 21 | (7.39) | 0.95 (0.37-2.43) |  |
| **Anthropometric measures** |  |  |  |  |  |  |
| **Pre-pregnancy BMI–** n (%) |  |  |  |  |  |  |
| Underweight or normal (BMI <25 kg/m^2^) | 57 | (74.03) | 193 | (68.68) | 1 |  |
| Overweight (25≤ BMI <30 kg/m^2^) | 8 | (10.39) | 65 | (23.13) | 2.40 (1.09-5.30) |  |
| Obese (BMI ≥30 kg/m^2^) | 12 | (15.58) | 23 | (8.19) | 0.57 (0.27-1.21) |  |
| **BMI at 1^st^ trimester questionnaire–** n (%) |  |  |  |  |  |  |
| Underweight or normal (BMI <25 kg/m^2^) | 53 | (67.95) | 180 | (64.98) | 1 | 1 |
| Overweight (25≤ BMI <30 kg/m^2^) | 11 | (14.10) | 69 | (24.91) | 1.85 (0.91-3.74) | 1.25 (0.55-2.84) |
| Obese (BMI ≥30 kg/m^2^) | 14 | (17.95) | 28 | (10.11) | 0.59 (0.29-1.20) | 0.62 (0.24-1.61) |
| **Weight gain between pre-pregnancy and 1^st^ trimester interview– kg** (mean ± SD) | 2.06 | ± 2.10 | 1.73 | ± 2.67 | 0.95 (0.85-1.05) | 1.02 (0.89-1.18) |

NVP = Nausea and vomiting of pregnancy; RAMQ = Régie de l’Assurance Maladie du Québec; BMI = Body mass index.

^a^ Adjusted for all variables of this column for which an adjusted OR is presented; shaded areas corresponds to estimates for which the univariate p value was > 0.15.

**^b^** Including gastroenteritis, motion sickness, [and food poisoning](javascript:affichage('1','17028758','ENG','','1')).

**^c^** Including asthma, anemia, depression, hypothyroidism, diabetes, epilepsy, hypertension and various problems like infections, eczema, migraines etc.

**^d^** Including folic acid, multivitamins, prenatal vitamins and iron.
