# Supplementary material for: Epidemiology of nausea and vomiting of pregnancy: prevalence, severity, determinants, and the importance of race/ethnicity
Source: BMC Pregnancy Childbirth. 2009 Jul 2;9:26. doi: 10.1186/1471-2393-9-26 (PMC2713199; doi:10.1186/1471-2393-9-26)
Supplement: Additional file 2 — Table of the determinants of NVP severity in the 1st trimester of pregnancy. Multivariate analyses showed that being born outside Canada, using medications to ease NVP, using non-pharmacological methods to ease NVP, and parity, were significantly associated with more severe NVP symptoms during the 1st trimester of pregnancy. Race/ethnicity was not found to be associated with NVP severity in the 1st trimester of pregnancy. [file 1471-2393-9-26-S2.doc]

**Additional file 2 - Determinants of NVP severity in the 1^st^ trimester of pregnancy.**

| **Characteristics** | **Mild NVP**  **n=145** | | **Moderate NVP**  **n = 126** | | **Severe NVP**  **n = 7** | | **Crude OR**  **(95% CI)** | **Adjusted OR ^a^**  **(95% CI)** |
| --- | --- | --- | --- | --- | --- | --- | --- | --- |
| **Socio-demographics characteristics** |  |  |  |  |  |  |  |  |
| **Maternal age -yr** (mean ± SD) | 31.35 | ± 4.50 | 32.31 | ± 4.67 | 31.43 | ± 3.31 | 1.04 (0.99-1.10) | 1.08 (0.99-1.17) |
| **Gestational age – wk** (mean ± SD) | 10.92 | ± 1.88 | 11.10 | ± 1.64 | 10.00 | ± 1.15 | 1.03 (0.90-1.17) | 1.00 (0.83-1.20) |
| **Country of birth –** n (%) |  |  |  |  |  |  |  |  |
| Canada | 107 | (73.79) | 76 | (60.32) | 2 | (28.57) | 1 |  |
| Other | 38 | (26.21) | 50 | (39.68) | 5 | (71.43) | 2.10 (1.27-3.45) | 2.97 (1.23-7.15) |
| **Race/ethnicity –** n (%) |  |  |  |  |  |  |  |  |
| Caucasian | 123 | (84.83) | 105 | (83.33) | 5 | (71.43) | 1 | 1 |
| Asian | 4 | (2.76) | 4 | 3.17) | 0 | (0.00) | 1.07 (0.27-4.30) | 0.73 (0.06-9.55) |
| Black | 11 | (7.59) | 11 | (8.73) | 2 | (28.57) | 1.49 (0.65-3.42) | 0.68 (0.16-2.93) |
| Hispanic | 7 | (4.83) | 6 | (4.76) | 0 | (0.00) | 0.92 (0.30-2.81) | 0.52 (0.11-2.39) |
| **Rx insurance plan –** n (%) |  |  |  |  |  |  |  |  |
| Provincial plan (RAMQ) only | 36 | (25.17) | 37 | (29.37) | 2 | (28.57) | 1 | 1 |
| Other insurance | 107 | (74.83) | 89 | (70.63) | 5 | (71.43) | 0.82 (0.48-1.38) | 1.85 (0.68-5.04) |
| **Work status –** n (%) |  |  |  |  |  |  |  |  |
| Student or not working | 30 | (20.83) | 35 | (27.78) | 2 | (28.57) | 1 | 1 |
| Working | 114 | (79.17) | 91 | (72.22) | 5 | (71.43) | 0.69 (0.40-1.19) | 1.21 (0.50-2.98) |
| **Living arrangement –** n (%) |  |  |  |  |  |  |  |  |
| With spouse or with someone (family or cotenant) | 143 | (98.62) | 121 | (96.80) | 7 | (100.00) | 1 | 1 |
| Living alone | 2 | (1.38) | 4 | (3.20) | 0 | (0.00) | 1.94 (0.38-9.82) | 4.29 (0.44-41.34) |
| **Education level–** n (%) |  |  |  |  |  |  |  |  |
| University completed | 97 | (67.36) | 83 | (65.87) | 2 | (28.57) | 1 | 1 |
| University not completed | 47 | (32.64) | 43 | (34.13) | 5 | (71.43) | 1.25 (0.77-2.05) | 1.64 (0.79-3.42) |
| **Household income – cdn$/yr** n (%) |  |  |  |  |  |  |  |  |
| Less than 40 000$ | 42 | (29.79) | 36 | (29.51) | 3 | (42.86) | 1 | 1 |
| Between 40 000 and 79 999$ | 32 | (22.70) | 34 | (27.87) | 3 | (42.86) | 1.25 ( 0.66-2.35) | 1.49 (0.54-4.11) |
| 80 000 and over $ | 67 | (47.52) | 52 | (42.62) | 1 | (14.29) | 0.81 (0.47-1.43) | 0.64 (0.20-2.05) |
| **Lifestyle habits** |  |  |  |  |  |  |  |  |
| **Exercise during 1^st^ trimester –** n (%) | 62 | (43.06) | 40 | (32.00) | 2 | (28.57) | 0.62 (0.38-1.01) | 0.76 (0.39-1.49) |
| **Smoking before pregnancy–** n (%) | 21 | (14.48) | 12 | (9.52) | 3 | (42.86) | 0.86 (0.43-1.72) | 2.11 (0.62-7.18) |
| **Smoking during 1^st^ trimester –** n (%) | 4 | (2.76) | 4 | (3.17) | 1 | (14.29) | 1.66 (0.44-6.20) | 2.36 (0.27-20.71) |
| **Coffee drinking before pregnancy–** n (%) | 123 | (84.83) | 97 | (76.98) | 6 | (85.71) | 0.64 (0.35-1.17) | 0.48 (0.19-1.27) |
| **Coffee drinking during 1^st^ trimester –** n (%) | 95 | (65.52) | 63 | (50.40) | 2 | (28.57) | 0.50 (0.31-0.80) | 0.66 (0.32-1.39) |
| **Use of alcohol before pregnancy –** n (%) | 104 | (71.72) | 74 | (58.73) | 6 | (85.71) | 0.64 (0.39-1.05) | 0.85 (0.38-1.88) |
| **Use of alcohol during 1^st^ trimester –** n (%) | 14 | (9.66) | 6 | (4.80) | 1 | (14.29) | 0.56 (0.22-1.40) | 0.64 (0.19-2.15) |
| **Health status and medications** |  |  |  |  |  |  |  |  |
| **Medications use to treat NVP in 1^st^ trimester –** n (%) | 16 | (11.19) | 37 | (29.37) | 3 | (42.86) | 3.37 (1.82-6.24) | 4.31 (1.96-9.45) |
| **Non-pharmacological methods use to treat NVP in 1^st^ trimester –** n (%) | 18 | (12.59) | 29 | (23.02) | 2 | (28.57) | 2.09 (1.13-3.89) | 6.63 (3.01-14.59) |
| **Infections or another situation causing nausea and/or vomiting in the 1^st^ trimester ^b^–** n (%) | 20 | (13.99) | 24 | (19.20) | 2 | (28.57) | 1.54 (0.82-2.89) | 1.21 (0.53-2.78) |
| **Comorbidities before pregnancy ^c^ –** n (%) |  |  |  |  |  |  |  |  |
| 0 | 97 | (66.90) | 98 | (77.78) | 5 | (71.43) | 1 |  |
| 1 | 41 | (28.28) | 25 | (19.84) | 2 | (28.57) | 0.64 (0.37-1.11) |  |
| 2 or 3 | 7 | (4.83) | 3 | (2.38) | 0 | (0.00) | 0.40 (0.10-1.59) |  |
| **Vitamin use before pregnancy ^d^–** n (% | 59 | (40.69) | 44 | (34.92) | 2 | (28.57) | 0.77 (0.47-1.24) | 1.27 (0.65-2.49) |
| **Vitamin use during 1^st^ trimester ^d^–** n (%) | 129 | (88.97) | 97 | (78.23) | 4 | (57.14) | 0.40 (0.21-0.75) | 0.57 (0.24-1.36) |
| **Oral contraceptives use in the last 6 months before pregnancy –** n (%) |  |  |  |  |  |  |  |  |
| No | 118 | (82.52) | 110 | (88.00) | 5 | (71.43) | 1 | 1 |
| Yes | 25 | (17.48) | 15 | (12.00) | 2 | (28.57) | 0.74 (0.38-1.43) | 0.70 (0.27-1.79) |
| **Pregnancy history** |  |  |  |  |  |  |  |  |
| **Gravidity –** n (%) |  |  |  |  |  |  |  |  |
| Multigravida | 114 | (78.62) | 113 | (89.68) | 7 | (100.00) | 1 |  |
| Primigravida | 31 | (21.38) | 13 | (10.32) | 0 | (0.00) | 0.39 (0.20-0.78) |  |
| **Parity –** n (%) |  |  |  |  |  |  |  |  |
| 0 | 45 | (31.47) | 18 | (14.29) | 1 | (14.29) | 1 | 1 |
| 1 | 77 | (53.85) | 68 | (53.97) | 3 | (42.86) | 2.16 (1.16-4.03) | 2.35 (0.99-5.55) |
| 2 or more (max: 4) | 21 | (14.69) | 40 | (31.75) | 3 | (42.86) | 4.80 (2.30-10.02) | 6.92 (2.47-19.36) |
| **Infertility experience –** n (%) | 10 | (6.90) | 10 | (8.06) | 0 | (0.00) | 1.05 (0.43-2.59) |  |
| **Anthropometric measures** |  |  |  |  |  |  |  |  |
| **Pre-pregnancy BMI–** n (%) |  |  |  |  |  |  |  |  |
| Underweight or normal (BMI <25 kg/m^2^) | 99 | (69.23) | 82 | (67.21) | 5 | (83.33) | 1 |  |
| Overweight (25≤ BMI <30 kg/m^2^) | 32 | (22.38) | 31 | (25.41) | 0 | (0.00) | 1.04 (0.59-1.84) |  |
| Obese (BMI ≥30 kg/m^2^) | 12 | (8.39) | 9 | (7.38) | 1 | (16.67) | 0.98 (0.41-2.37) |  |
| **BMI at 1^st^ trimester questionnaire –** n (%) |  |  |  |  |  |  |  |  |
| Underweight or normal (BMI <25 kg/m^2^) | 93 | (66.91) | 79 | (64.75) | 3 | (50.00) | 1 | 1 |
| Overweight (25≤ BMI <30 kg/m^2^) | 30 | (21.58) | 33 | (27.05) | 2 | (33.33) | 1.34 (0.76-2.36) | 0.83 (0.38-1.80) |
| Obese (BMI ≥30 kg/m^2^) | 16 | (11.51) | 10 | (8.20) | 1 | (16.67) | 0.81-0.36-1.84) | 0.59 (0.18-1.87) |
| **Weight gain between pre-pregnancy and 1^st^ trimester interview– kg** (mean ± SD) | 1.66 | ± 3.10 | 1.61 | ± 2.04 | 3.68 | ± 3.26 | 1.02 (0.93-1.11) | 0.93 (0.81-1.08) |

NVP = Nausea and vomiting of pregnancy; RAMQ = Régie de l’Assurance Maladie du Québec; BMI = Body mass index.

^a^ Adjusted for all variables of this column for which an adjusted OR is presented; shaded areas corresponds to estimates for which the univariate p value was > 0.15.

**^b^** Including gastroenteritis, motion sickness, [and food poisoning](javascript:affichage('1','17028758','ENG','','1')).

**^c^** Including asthma, anemia, depression, hypothyroidism, diabetes, epilepsy, hypertension and various problems like infections, eczema, migraines etc.

**^d^** Including folic acid, multivitamins, prenatal vitamins and iron.
